# Expression of CHPF modulates cell proliferation and invasion in lung cancer

**DOI:** 10.1590/1414-431X20209021

**Published:** 2020-04-27

**Authors:** Chengsong Cao, Yong Liu, Qun Wang, Jing Zhao, Ming Shi, Junnian Zheng

**Affiliations:** 1 Department of Oncology, Nanjing Medical University, Nanjing, Jiangsu, China; 2 Department of Oncology, Xuzhou Center Hospital, Xuzhou, Jiangsu, China; 3 Department of Oncology, Xuzhou Medical University, Xuzhou, Jiangsu, China; 4 Xuzhou Institute of Medical Science, Xuzhou, Jiangsu, China

**Keywords:** CHPF, Lung cancer, Proliferation, Invasion, TCGA, Affymetrix Gene Chip

## Abstract

Lung cancer is the most common malignancy worldwide and is characterized by rapid progression, aggressive behavior, frequent recurrence, and poor prognosis. The TCGA database indicates that chondroitin polymerizing factor (*CHPF*) is overexpressed in human lung cancer tissues compared with normal tissues and this overexpression corresponds to shorter overall survival in lung cancer patients. In this study, to investigate the function of *CHPF* in lung cancer, lentiviral vectors expressing *CHPF* shRNA were stably transduced into A549 and H1299 cells. Compared to shCtrl cells, *CHPF* knockdown cells had significantly reduced proliferation. Furthermore, the silencing of *CHPF* in A549 and H1299 cells resulted in apoptotic induction, which led to decreased colony formation. Wound healing and transwell invasion assays revealed that *CHPF* could positively regulate the migration of lung cancer cells. The tumorigenic role of *CHPF* was also validated in nude mouse xenograft models. Affymetrix gene chip analysis indicated that *CHPF* regulated the proliferation and invasion of lung cancer cells through *CDH1*, *RRM2*, *MKI67*, and *TNFRSF10B*. We thus highlight *CHPF* as a novel target for lung cancer treatment.

## Introduction

Lung cancer is one of the most lethal and common malignancies in humans, with a 5-year survival rate of ≤20% (1,2). This poor survival rate is related to an unclear pathogenesis and a lack of effective early diagnosis and treatment methods (3,4). Knowledge of the molecular and cellular mechanisms that govern lung cancer development can improve future diagnostics and therapies (5–7). In recent years, kinase-targeted therapies and immune check-point inhibitors have shown increased efficacy in lung cancer treatment compared to standard chemotherapy (6,8,9). However, some problems persist, including specificity, cell penetrability, and resistance. More specifically, characterized drug targets are required to overcome these barriers.

Chondroitin polymerizing factor (*CHPF*) is a 775 amino acid type II transmembrane protein and a member of the chondroitin synthase family (10–12). The *CHPF* gene is located on region 2q35-q36 of the human chromosome, spanning 4 exon regions. Studies have shown that *CHPF* expression is upregulated in colorectal cancer (13), laryngeal cancer (14), and brain glioma (15) and that the overexpression of *CHPF* may be closely related to tumor occurrence and development. However, the mechanism of high *CHPF* expression in lung cancer development and progression has not been studied in detail. An in-depth knowledge of the molecular mechanism and related signaling pathways that govern *CHPF* activity may be of benefit in lung cancer treatment.

In this study, we demonstrated elevated expression of *CHPF* mRNA in lung cancer tissues and five lung cancer cell lines. The effects of *CHPF* on the proliferation and invasion of lung cancer cells were further assessed *in vitro* and *in vivo*. Microarray analysis of the gene expression changes in *CHPF*-silenced cells was performed to reveal the pathways regulated by *CHPF*.

## Material and Methods

### Cell lines and cell culture

The human lung cancer cell lines A549, 95-D, NCI-H1299, H1688, and NCI-H460 were purchased from the Type Culture Collection of the Chinese Academy of Sciences (China). Cells were cultured in F-12K complete medium (ATCC) containing 10% fetal bovine serum (Invitrogen, USA) at 37°C with 5% CO_2_.

### TCGA database analysis

The RNA-sequencing dataset of the lung cancer cohort was downloaded from TCGA database (https://tcga-data.nci.nih.gov/tcga/). For lung adenocarcinoma (LUAD), the expression data for *CHPF* in 57 paired (tumor and peri-tumor) samples and in normal (n=59) and primary tumor tissues (n=515) were collected and analyzed. Additionally, the survival of LUAD patients with low/medium (n=375) and high expression (n=127) of *CHPF* was statistically analyzed.

### RNA isolation and quantitative real-time PCR (qRT-PCR)

Total RNA was isolated from five lung cancer cell lines (A549, 95-D, NCI-H1299, H1688, and NCI-H460) using TRIzol total RNA reagent (Pufei Biotech, China). Reverse transcription was conducted according to the instructions of M-MLV reverse transcriptase (Promega, USA) to obtain cDNA. The primers for *CHPF* were synthesized by Gene Chem Co. Ltd. (China). GAPDH was applied as a loading control. The sequences of the primers used in the study are as follows: GAPDH forward, 5′-TGACTTCAACAGCGACACCCA-3′ and reverse, 5′-CACCCTGTTGCTGTAGCCAAA-3′; *CHPF* forward, 5′-GGAACGCACGTACCAGGAG-3′ and reverse, 5′-CGGGATGGTGCTGGAATACC-3′. The reactions were performed using SYBR premix Ex Taq II (Takara Biomedical Technology Co., Ltd., Japan). Relative *CHPF* expression was analyzed by normalizing to GAPDH. The comparative threshold cycle (2^-△△Ct^ and 10000/2^△Ct^) equation was applied to calculate the relative *CHPF* mRNA expression.

### shRNA lentiviral vector construction and transduction

To silence *CHPF*, cells were transduced with short hairpin (shRNA) lentivirus targeting the human *CHPF* gene (Gene ID: 79586) with pGCSIL-green fluorescent protein (GFP) for transduction rate evaluation. The shRNA sequence was as follows: shRNA-*CHPF* 5′-CTGGCCATGCTACTCTTTG-3′. Lentivirus lacking the shRNA insert was used as a control. A549 and H1299 cells were seeded into a 6-well plate at a density of 4×10^5^ cells/well and transduced with shRNA-*CHPF* (6×10^8^ TU/mL) or shRNA-NC lentivirus (8×10^8^ TU/mL). After 72 h of transduction, the cells were imaged under a fluorescence microscope and further selected by puromycin. Five days post-infection, *CHPF* silencing was verified through qRT-PCR analysis.

### Western blotting

The cells were lysed with RIPA buffer for 30 min at 4°C for protein extraction after infection with lentivirus. A BCA assay was applied to determine the protein concentrations. The same amounts of protein were separated on 12.5% SDS-PAGE gels and transferred to polyvinylidene fluoride (PVDF) membranes. The membranes were incubated with anti-*CCND1* (#2978) or anti-*CDH1* (#14472) primary antibodies (Cell Signaling Technologies (CST), USA) as well as other antibodies, including those against *MKI67* (ab15580), *TNFRSF10B* (ab8416), *FOXM1* (ab180710), *RRM2* (ab172476), *HIF1A* (ab16066) (Abcam, UK), and *GAPDH* (SC-32233) (Santa Cruz Biotechnology, USA). Anti-*CHPF* antibody (Orb127868) was purchased from Biorbyt Ltd. (UK). The membranes were then incubated with HRP-conjugated antibodies (CST, #7076, #7074).

### MTT assays

After infection with shCtrl or sh*CHPF* lentivirus, 1.5×10^3^ A549 and H1299 cells were seeded into 96-well plates and further cultured at 37°C for 1–5 days. Cells were counted using the Cellomics ArrayScan VT1 HCS automated reader (Cellomics, Inc., USA). Cell proliferation was determined by MTT assay according to the manufacturer's protocol. Briefly, after the incubation of MTT reagent with cells for 4 h, absorbance was read at 490 nm on the microplate reader.

### Apoptosis assays

The cells infected with shCtrl or sh*CHPF* lentivirus were collected and labelled with annexin V-APC according to the manufacturer's protocol (eBioscience, USA). Annexin staining was measured on a FACS Calibur II sorter, and Cell Quest Research software (BD Biosciences, USA) was used for analysis.

### Colony forming assays

Soft agar assays were used to assess the regulation of colony formation by *CHPF* at 10 days post-infection. Colonies were fixed in 4% PFA and Giemsa-stained (Sigma-Aldrich, USA). Colonies larger than 100 μm were counted.

### Invasion assays

Transwell membranes pre-coated with Matrigel (BD Biosciences) were applied to evaluate the invasion effect mediated by *CHPF*. A total of 8×10^4^ cells were seeded into the insert and the lower chamber of the Transwell was filled with 500 μL of F-12K (Invitrogen, USA) supplemented with 10% FBS. After incubation for 24 h, the invading cells were stained with Giemsa after the cells in the upper chamber were removed.

### Cell migration assays

A wound healing assay was used to determine the migration of the cells. A total of 5×10^4^ cells were seeded into 96-well plates and grown to 80-90% confluency. A scratch line was made in the cell monolayer by a pipette tip. Cells were further cultured for different periods of time as indicated. The migrated cells were quantified using Celigo (Nexcelom, USA), and the migration rate was calculated.

### Xenograft models

The animal experiment was approved by the Ethics Committee of Nanjing Medical University (No. IACUC-1808010). Four-week-old female BALB/c nude mice, which were bought from Ling Chang (China), were randomly divided into two groups (n=6 per group). shRNA-*CHPF* or normal control (NC) lentivirus-expressing A549 cells (1×10^7^) were subcutaneously implanted into the right dorsal flank. The tumor volume was measured twice weekly with calipers and calculated using the following formula: V = 3.14 / 6 × length × width^2^. Forty days post-inoculation, the tumors were excised and weighed.

### Microarray and data analysis

Total RNA was extracted and purified with clean-up kits (Pufei, China). The Affymetrix Gene Chip Human Transcriptome Array 2.0 (Affymetrix Inc., USA) was used for microarray hybridization, and the chip was scanned. The signals were converted into digital information and analyzed by SAM software (Affymetrix Inc., USA). The Empirical Bayes procedure and Benjamini-Hochberg method were used to calculate and correct the P-value. Genes with a corrected P-value less than 0.05 and an absolute value of fold-change greater than or equal to 2 were considered to be significantly differentially expressed. GO enrichment analysis of A549 cells was performed to explore the biological function of *CHPF* using the R software. Ingenuity Pathway Analysis (Ingenuity Systems, Inc., USA, http://www.ingenuity.com) was used to explore protein networks.

### Statistical analysis

Data are reported as means±SD of three replicates. Statistical analysis was carried out using the SPSS 20.0 statistical software package (IBM, USA) and Student's *t*-test. Statistical significance was accepted at P-value <0.05.

## Results

### *CHPF* was overexpressed in lung cancer tissues and cells

**Figure 1:**
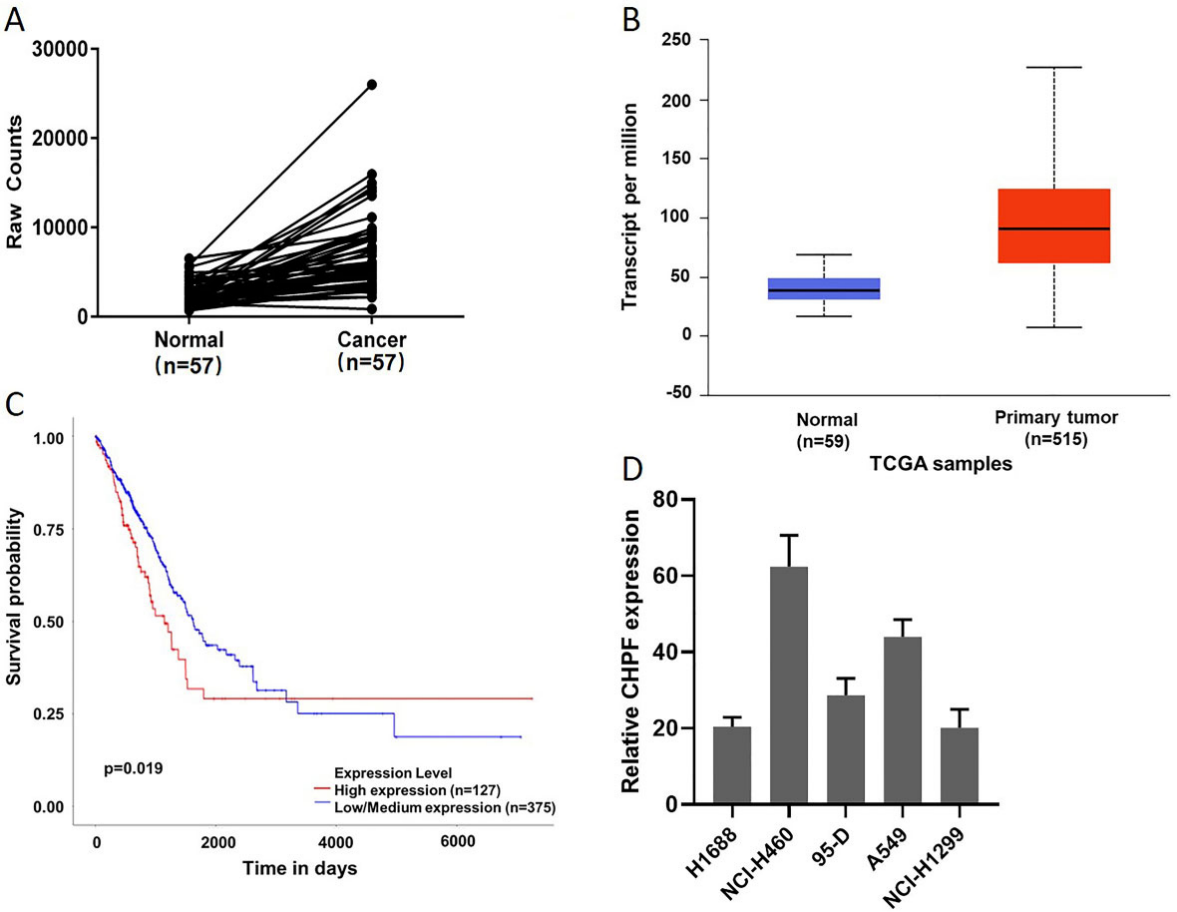
Chondroitin polymerizing factor (*CHPF*) expression in lung cancer patients and lung cancer cell lines. **A**, *CHPF* mRNA expression in the lung cancer and normal tissues of samples. The solid line that shows the trend in gene expression. **B**, *CHPF* expression in human lung cancer compared to normal tissue in the TCGA database. **C**, Elevated *CHPF* expression corresponded to shorter overall survival of lung adenocarcinoma (LUAD) patients from the TCGA database. **D**, *CHPF* mRNA expression of five lung cancer cell lines was detected by qRT- PCR and calculated by the 10000/2^△Ct^ equation. Data are reported as median and interquartile range (**B**) and means±SD (**D**).

Through the analysis of the datasets from TCGA, it was found that, compared with human normal tissues, lung cancer tissues had significant overexpression of *CHPF* mRNA (Figure 1A and B, Supplementary Table S1). Elevated *CHPF* expression corresponded to shorter overall survival in lung cancer patients (Figure 1C). *CHPF* mRNA levels were then assessed in lung cancer cell lines including A549, 95-D, NCI-H1299, H1688, and NCI-H460 by qRT-PCR. The results confirmed that *CHPF* mRNA was expressed at high levels in all five cell lines (Figure 1D).

### *CHPF* silencing inhibited lung cancer cell proliferation

**Figure 2:**
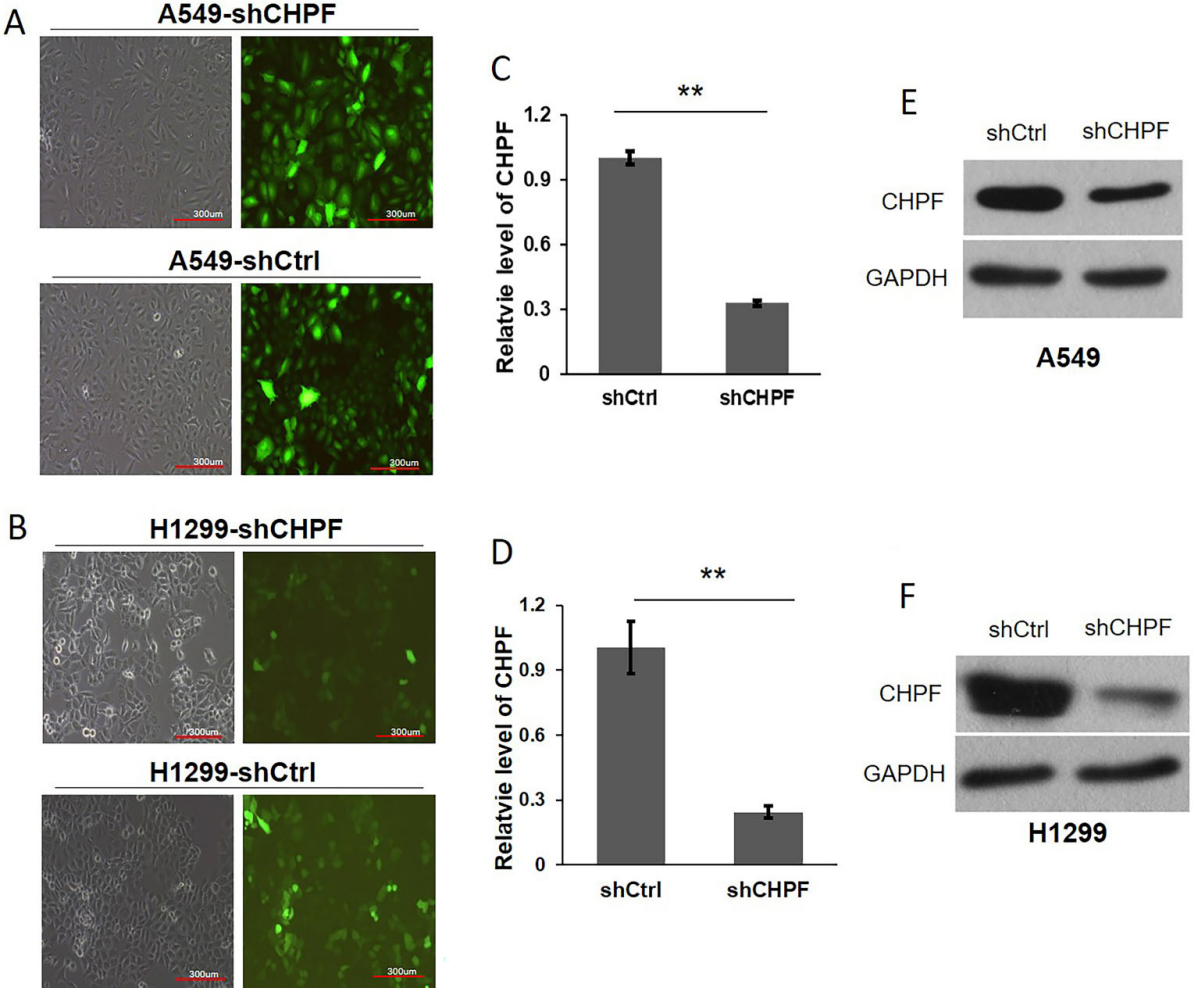
shRNA lentivirus transduction and confirmation. **A** and **B**, Lentiviral vector expressing sh*CHPF* or shCtrl was transduced into A549 and H1299 cells. Infection efficiency was confirmed by fluorescence microscopy at 72 h. **C** to **F**, qRT-PCR and western blotting were used to confirm the knockdown efficiency of *CHPF*. **P<0.01 (Student's *t*-test). *CHPF*: chondroitin polymerizing factor.

**Figure 3:**
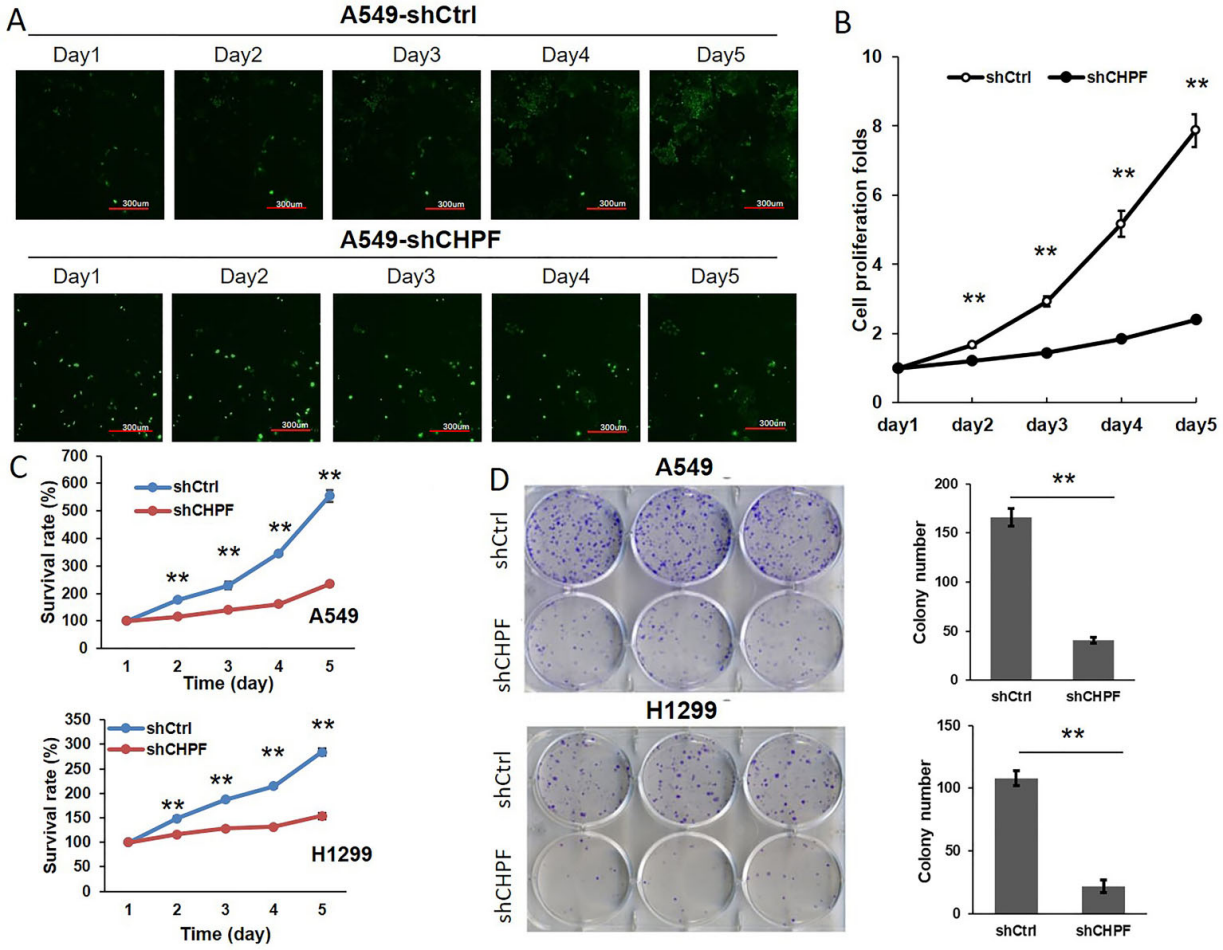
Effect of chondroitin polymerizing factor (*CHPF*) on the proliferation and colony formation of lung cancer cells. **A** and **B**, High-content cell imaging was used daily to assess cell growth in A549 cells following lentivirus infection (scale bar: 300 μm). **C**, MTT assays displaying the survival of A549 and H1299 cells after infection with sh*CHPF* or shCtrl for 5 days. **D**, Impact of *CHPF* silencing on the colony formation of A549 and H1299 lung cancer cells. Data are reported as means±SD. **P<0.01 (Student's *t*-test).

More than 80% of the A549 and H1299 cells that were infected with shCtrl or sh*CHPF* lentivirus at 6×10^8^ TU/mL exhibited green fluorescence, indicating successful lentiviral infection (Figure 2A and B). Lentivirus-mediated RNA interference of *CHPF* expression was confirmed by qRT-PCR (Figure 2C and D) and western blotting (Figure 2E and F). To explore the impact of *CHPF* on cell proliferation, A549 cells expressing sh*CHPF* or shCtrl lentivirus were seeded into 96-well plates and Cellomics was used to analyze cell proliferation (Figure 3A). Two days post-seeding, the proliferation rates of sh*CHPF*-infected cells were significantly lower than those of shCtrl cells in a time-dependent manner (Figure 3B). Thus, *CHPF* silencing significantly inhibited A549 cell growth. The suppression effect of *CHPF* silencing on cell growth in A549 and H1299 cells was further determined by MTT assay in a five-day culture. As shown in Figure 3C, cell survival of both A549 and H1299 cells was significantly decreased, starting on the second day in the *CHPF*-silenced groups (P<0.01). The ability to form colonies is a characteristic of malignant tumor cells. Giemsa staining was performed to explore the effect of *CHPF* on the colony forming ability of lung cancer cells after 15 days of culture. The number of colonies formed by A549 cells infected with sh*CHPF* was significantly lower than the number formed by the shCtrl cells (41±3 *vs* 166±9). Colony forming ability was also significantly lower in H1299 cells in the sh*CHPF* group compared to the shCtrl control group (22±5 *vs* 108±6, P<0.01; Figure 3D).

### CHPF silencing induced cell apoptosis

**Figure 4:**
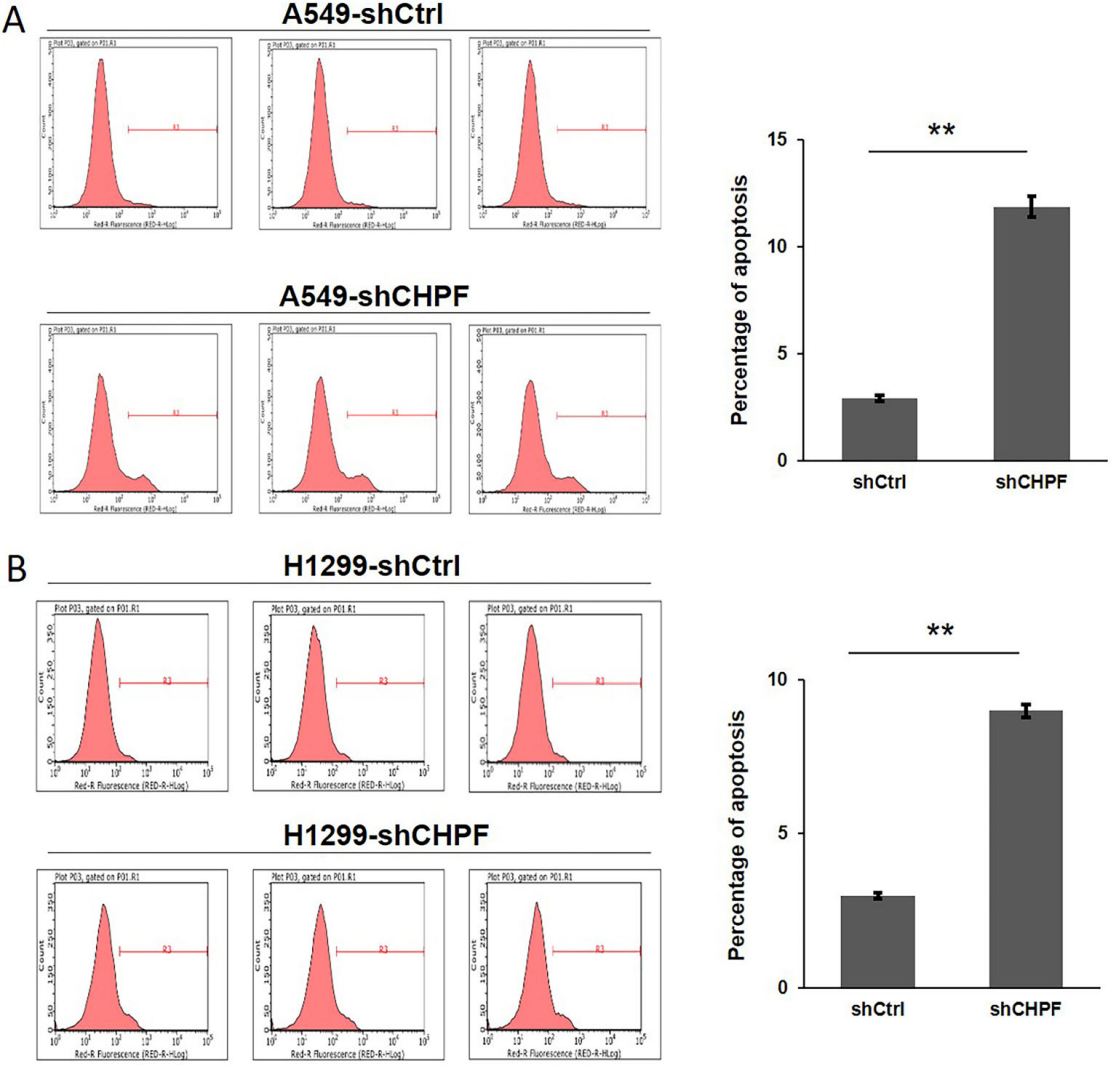
Effect of chondroitin polymerizing factor (*CHPF*) on the apoptosis of lung cancer cells. **A** and **B**, The apoptotic rates of A549 and H1299 cells after infection with shRNA-*CHPF* or shCtrl were analyzed by flow cytometry. Data are reported as means±SD of three independent experiments. **P<0.01 (Student's *t*-test).

We further examined the effects of *CHPF* silencing on A549 and H1299 cell apoptosis by flow cytometry. The rate of apoptosis in the sh*CHPF* A549 cells (11.88±0.50%) was significantly higher than that in the shCtrl cells (2.91±0.14%), (P<0.01; Figure 4A). Similarly, the percentage of apoptotic H1299 cells in the sh*CHPF* group (8.99±0.21%) was higher than that in the shCtrl group (2.98±0.10%) (P<0.01; Figure 4B).

### Inhibiting *CHPF* repressed the invasion and migration abilities of lung cancer cells

**Figure 5:**
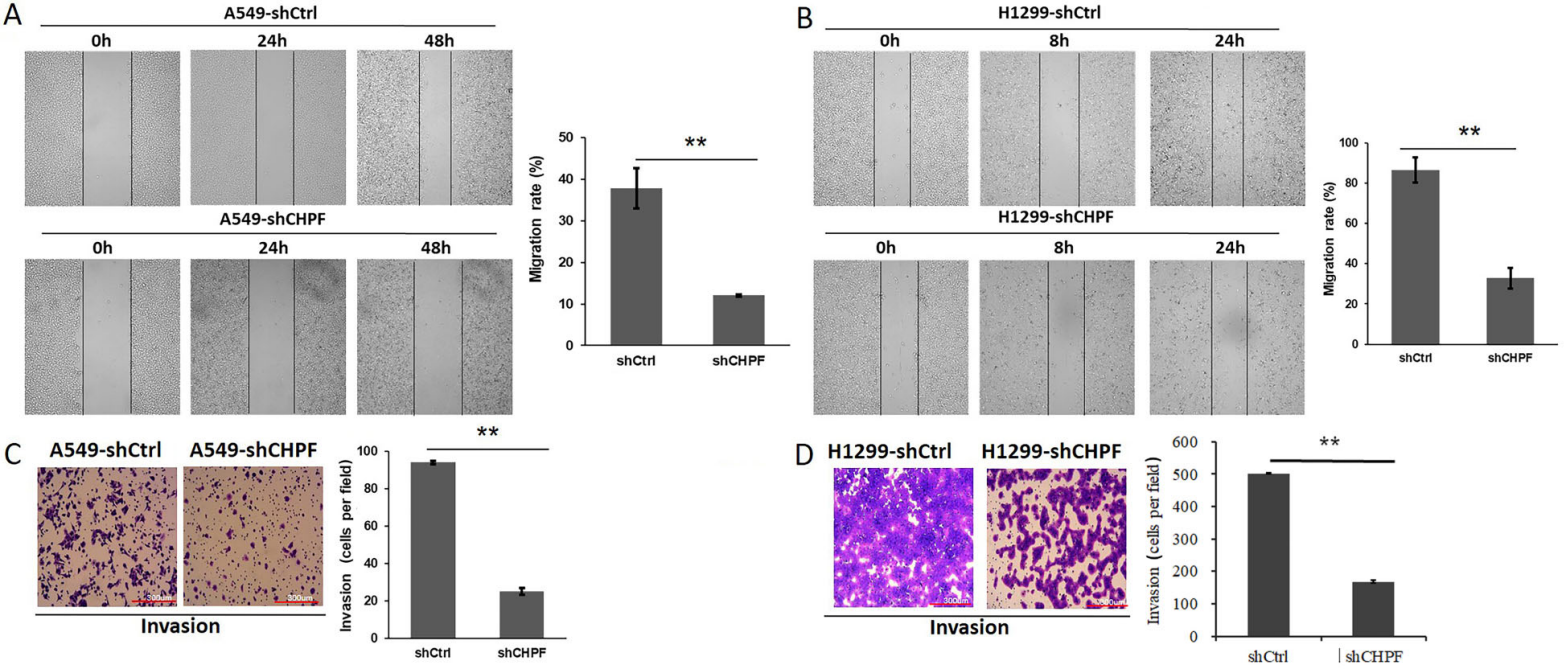
Effect of chondroitin polymerizing factor (*CHPF*) on the migration and invasion of A549 and H1299 cells. **A,** and **B,** A wound healing assay was applied to measure the number of migratory A549 and H1299 cells at the indicated time points. The cell invasive ability of A549 (**C**) and H1299 cells (**D**) was determined by the transwell assay after infection (scale bar: 300 μm). Data are reported as means±SD of three independent experiments. **P<0.01 (Student's *t*-test).

To evaluate the role of *CHPF* in lung cancer metastasis, we assessed the effects of *CHPF* silencing in A549 and H1299 cells. Wound healing assays showed that the motility of A549 cells in the sh*CHPF* group was significantly lower than that in the shCtrl control (12.03±0.32% *vs* 37.86±4.86%) (P<0.01; Figure 5A). The motility of H1299 cells in the sh*CHPF* group was significantly lower than that in the shCtrl group (32.84±5.07% *vs* 86.54±6.26%) (P<0.01; Figure 5B). Moreover, the invasive ability of both A549 and H1299 cells was significantly decreased following *CHPF* silencing as determined by the transwell assay (sh*CHPF*: 25±1.86 *vs* shCtrl: 94±0.89 for A549 cells, sh*CHPF:* 168±4.14 *vs* shCtrl: 503±1.98 for H1299 cells, P<0.01; Figure 5C and D). These data strongly support a role for *CHPF* in the migration and invasion of A549 and H1299 cells.

### *CHPF* silencing decreased tumorigenicity in nude mice

**Figure 6:**
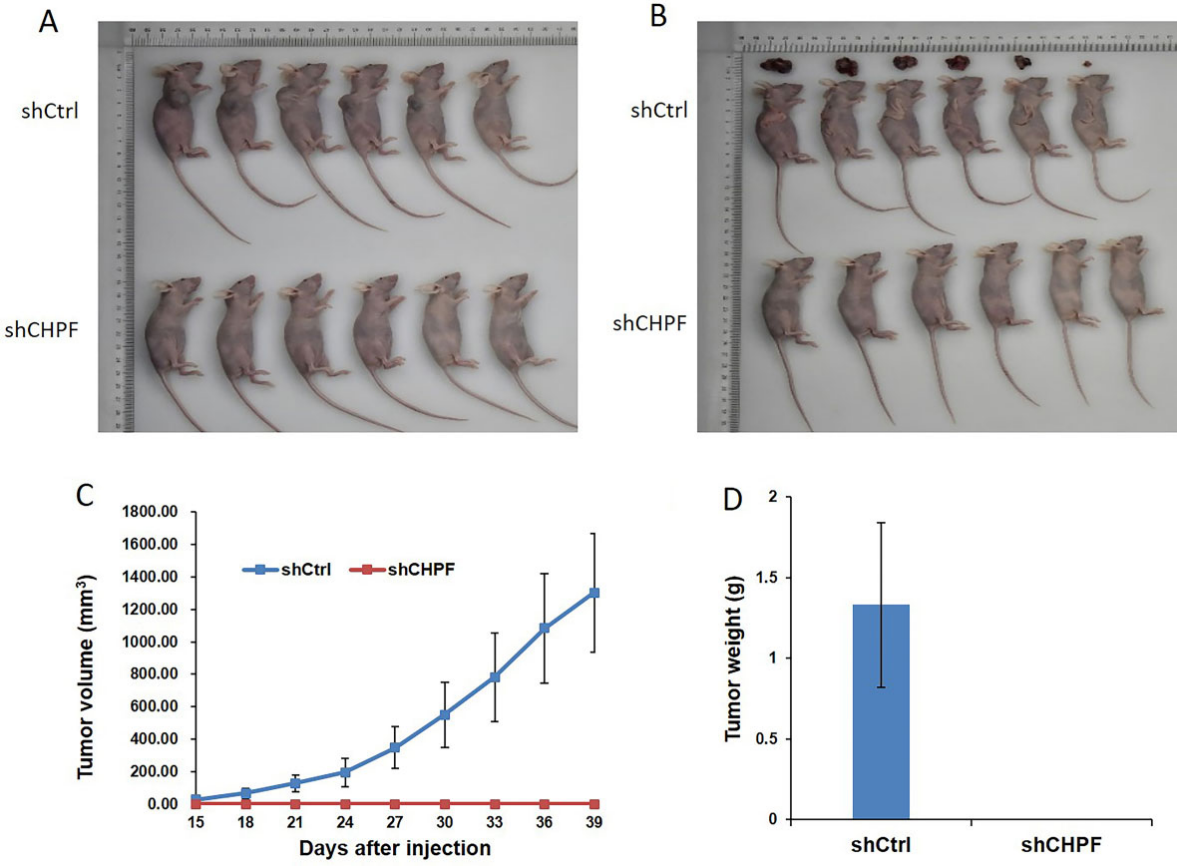
Effect of chondroitin polymerizing factor (*CHPF*) on tumorigenicity in nude mice. **A**, Equal numbers of CHPF silenced (sh*CHPF*) or control A549 cells were subcutaneously administered to nude mice. **B**, Excised tumors were recorded. The volume (**C**) and weight (**D**) of tumors were measured (n=6). Data are reported as means±SD.

A lung cancer xenograft mouse model was developed to validate the oncogenic role of *CHPF*. BALB/c nude mice were selected for subcutaneous injection of A549 cells infected with either shCtrl or sh*CHPF* lentivirus. Tumor growth was continuously followed after cell implantation. Tumors were excised and their size and weight were measured (Figure 6A and B). After 40 days, minimal tumor growth was observed in groups injected with *CHPF*-silenced cells, and tumor weights were significantly lower in the *CHPF*-silenced group than that in the control group (Figure 6C and D). These results clearly indicated that *CHPF* silencing inhibited lung cancer cell tumorigenicity *in vivo*.

### *CHPF* silencing altered oncogene expression in lung cancer cells

**Figure 7:**
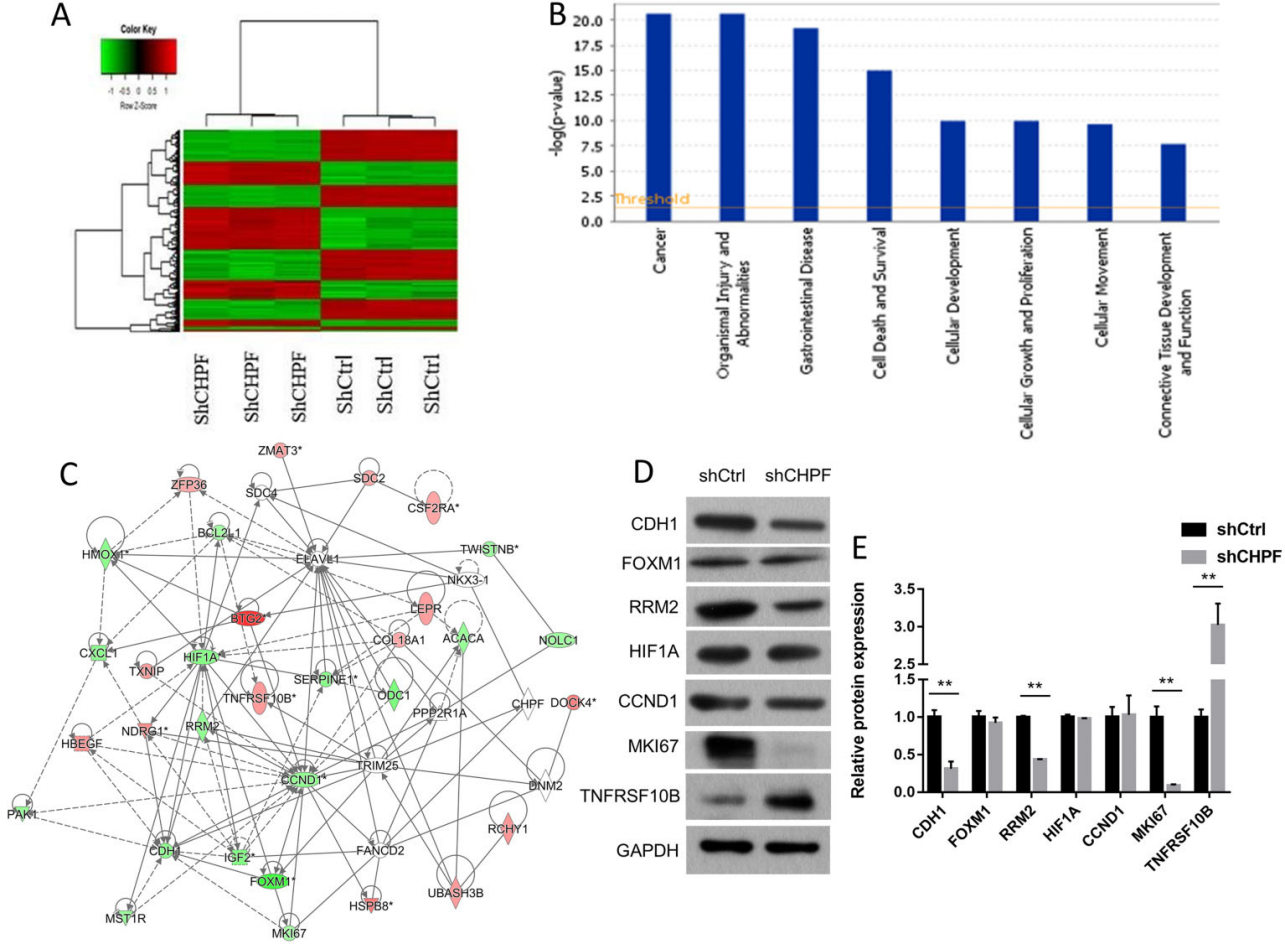
Effect of chondroitin polymerizing factor (*CHPF*) on global gene expression. **A**, Heatmap representation of 635 differentially expressed genes (DEGs) in A549 cells infected with sh*CHPF* or shCtrl. P<0.05 and fold-change >2 (red: upregulated genes; green: downregulated genes). **B**, Functional pathway enrichment of the DEGs was analyzed via Ingenuity Pathway Analysis. The x-axis represents the pathway. Statistical significance is shown on the y-axis and represents the inverse log of the P-value. **C**, The network was built on the basis of microarray data of lung cancer cells. Red represents upregulation and green represents downregulation. **D**, Western blotting analysis was applied to evaluate the expression of *CDH1*, FOXM1, *RRM2*, *HIF1A*, *CCND1*, *MKI67*, and *TNFRSF10B* in sh*CHPF* and shCtrl lung cancer cells. Data are reported as means±SD. **P<0.01 (Student's *t*-test).

Global gene expression profiling of A549 cells infected with lentivirus expressing either shCtrl or sh*CHPF* was examined using a microarray platform. Following normalization and bioinformatic analysis, 635 differentially expressed genes (DEGs) were identified (P<0.05 and absolute fold-change (FC absolute) >2), including 294 upregulated and 341 downregulated genes (Figure 7A). *CHPF* function and pathway enrichment analyses were performed using (Ingenuity Pathway Analysis). DEGs were significantly enriched in cancer, organismal injury, gastrointestinal disease, cell death, and other pathways (Figure 7B). The interaction network of *CHPF* with the identified cancer associated genes was then assessed (Figure 7C). In total, 32 genes which are related to cancer development were identified to be regulated by *CHPF*. Among the genes, *CCND1*, *MKI67*, *HIF1A*, *CDH1*, *RRM2*, and *FOXM1* were significantly downregulated following *CHPF* silencing (Supplementary Table S2). Western blot analysis confirmed decreased *CDH1*, *RRM2*, and *MKI67* expression in *CHPF* silenced cells. *TNFRSF10B* expression was significantly increased following *CHPF* silencing (Figure 7D).

## Discussion

*CHPF* is a member of the chondroitin synthase family and participates in the extension of the chondroitin sulfate (CS) backbone (16). The expression and activity of *CHPF* is indispensable for the initiation of CS biosynthesis and the production of proteoglycan. In recent years, *CHPF* has been implicated in colorectal cancer, laryngeal cancer, and glioma development (13–15). However, the function of *CHPF* in human lung cancer has remained undefined.

In this study *in vitro*, we found that *CHPF* silencing inhibited proliferation and colony formation and promoted cellular apoptosis in lung cancer cells. Moreover, *CHPF* silencing was also demonstrated to reduce lung cancer cell migration and invasion. The *in vivo* study revealed that *CHPF* contributed to the tumorigenicity of lung cancer cells in xenograft mouse models.

To investigate the molecular mechanism governing the ability of *CHPF* to promote tumorigenesis, Affymetrix gene chip analysis was used to explore changes in the expression of cancer-related genes in sh*CHPF* and shCtrl cells. In total, 32 cancer related genes including *CCND1*, *MKI67*, *HIF1A*, *CDH1*, *RRM2*, and *FOXM1* were subsequently identified through Ingenuity Pathway Analysis. CHPF silencing markedly downregulated the mRNA expressions of six cancer genes at the transcriptional level. The loss of expression of these genes was validated at the protein level by western blotting. *RRM2* is known to be associated with tumor invasion and with the establishment of a metastatic phenotype (17,18), which promotes poor overall and progression-free survival (19,20). In our study, it was validated that *CHPF* downregulation was associated with decreased *RRM2* levels.

The *MKI67* labeling index also correlates with tumor growth rates (21), histologic stage (22), and tumor recurrence (23). Elevated *MKI67* expression indicates rapid cancer progression and poor prognosis. According to our investigation, *CHPF* silencing decreased *MKI67* expression, which may contribute to the effects of *CHPF* depletion on malignancy. Death receptor (DR) signaling forms one arm of the extrinsic apoptotic processes (24). TNF-related apoptosis-inducing ligand (*TRAIL*) and its receptor DR5 (*TNFRSF10B*) promote tissue damage in somatic cells (25). It was suggested that *CHPF* silencing in A549 cells may enhance apoptosis through the upregulation of *TNFRSF10B*. The *CDH1* gene is believed to be related to tumor proliferation (26). The above data suggest that the effects of *CHPF* silencing on lung cancer cell proliferation may be related to the downregulation of *CDH1*. Additionally, using Ingenuity Pathway Analysis, we identified numerous genes regulated by *CHPF* centralized networks that need further investigation.

In summary, we found that the expression of *CHPF* in lung cancer tissues was higher than that in normal lung tissues. Elevated *CHPF* expression corresponded to shorter overall survival. *CHPF* was found to promote lung cancer cell proliferation, migration, and invasion *in vitro* and tumorigenesis *in vivo*. It is speculated that *CHPF* mediated the above effects through the dysregulation of cancer-related genes, including *CDH1*, *RRM2*, *MKI67*, and *TNFRSF10B*. These findings provide a proof-of-concept that revealed the cellular roles of *CHPF* and its contribution to lung cancer malignancy. This highlighted *CHPF* as a potential therapeutic target for the clinical treatment of lung cancer.

## Supplementary Material

Click here to view [pdf].

## References

[1] Siegel RL, Miller KD, Jemal A (2018). Cancer statistics, 2018. CA Cancer J Clin.

[2] Wong MCS, Lao XQ, Ho KF, Goggins WB, Tse SLA (2017). Incidence and mortality of lung cancer: global trends and association with socioeconomic status. Sci Rep.

[3] Testa U, Castelli G, Pelosi E (2018). Lung Cancers: molecular characterization, clonal heterogeneity and evolution, and cancer stem cells. Cancers (Basel).

[4] Tsvetkova E, Goss GD (2012). Drug resistance and its significance for treatment decisions in non-small-cell lung cancer. Curr Oncol.

[5] Hubers AJ, Prinsen CF, Sozzi G, Witte BI, Thunnissen E (2013). Molecular sputum analysis for the diagnosis of lung cancer. Br J Cancer.

[6] Politi K, Zakowski MF, Fan PD, Schonfeld EA, Pao W, Varmus HE (2006). Lung adenocarcinomas induced in mice by mutant EGF receptors found in human lung cancers respond to a tyrosine kinase inhibitor or to down-regulation of the receptors. Genes Dev.

[7] Chen Z, Fillmore CM, Hammerman PS, Kim CF, Wong KK (2014). Non-small-cell lung cancers: a heterogeneous set of diseases. Nat Rev Cancer.

[8] Roskoski R (2017). Anaplastic lymphoma kinase (ALK) inhibitors in the treatment of ALK-driven lung cancers. Pharmacol Res.

[9] Tanvetyanon T, Gray JE, Antonia SJ (2017). PD-1 checkpoint blockade alone or combined PD-1 and CTLA-4 blockade as immunotherapy for lung cancer?. Expert Opin Biol Ther.

[10] Siebert JR, Conta Steencken A, Osterhout DJ (2014). Chondroitin sulfate proteoglycans in the nervous system: inhibitors to repair. Biomed Res Int.

[B11] Kitagawa H, Uyama T, Sugahara K (2001). Molecular cloning and expression of a human chondroitin synthase. J Biol Chem.

[12] Kitagawa H, Izumikawa T, Uyama T, Sugahara K (2003). Molecular cloning of a chondroitin polymerizing factor that cooperates with chondroitin synthase for chondroitin polymerization. J Biol Chem.

[13] Kalathas D, Theocharis DA, Bounias D, Kyriakopoulou D, Papageorgakopoulou N, Stavropoulos MS (2011). Chondroitin synthases I, II, III and chondroitin sulfate glucuronyltransferase expression in colorectal cancer. Mol Med Rep.

[14] Kalathas D, Triantaphyllidou IE, Mastronikolis NS, Goumas PD, Papadas TA, Tsiropoulos G (2010). The chondroitin/dermatan sulfate synthesizing and modifying enzymes in laryngeal cancer: expressional and epigenetic studies. Head Neck Oncol.

[15] Fan YH, Xiao B, Lv SG, Ye MH, Zhu XG, Wu MJ (2017). Lentivirusmediated knockdown of chondroitin polymerizing factor inhibits glioma cell growth in vitro. Oncol Rep.

[16] Mizumoto S, Yamada S, Sugahara K (2014). Human genetic disorders and knockout mice deficient in glycosaminoglycan. Biomed Res Int.

[17] Reichard P (2002). Ribonucleotide reductases: the evolution of allosteric regulation. Arch Biochem Biophys.

[18] Tanaka H, Arakawa H, Yamaguchi T, Shiraishi K, Fukuda S, Matsui K (2000). A ribonucleotide reductase gene involved in a p53-dependent cell-cycle checkpoint for DNA damage. Nature.

[19] Liu X, Zhang H, Lai L, Wang X, Loera S, Xue L (2013). Ribonucleotide reductase small subunit M2 serves as a prognostic biomarker and predicts poor survival of colorectal cancers. Clin Sci (Lond).

[20] Farrell JJ, Moughan J, Wong JL, Regine WF, Schaefer P, Benson AB 3rd (2016). Precision medicine and pancreatic cancer: a gemcitabine pathway approach. Pancreas.

[21] Maeda T, Takenaka K, Adachi E, Matsumata T, Shirabe K, Honda H (1995). Small hepatocellular carcinoma of single nodular type: a specific reference to its surrounding cancerous area undetected radiologically and macroscopically. J Surg Oncol.

[22] Ng IO, Na J, Lai EC, Fan ST, Ng M (1995). Ki-67 antigen expression in hepatocellular carcinoma using monoclonal antibody MIB1. A comparison with proliferating cell nuclear antigen. Am J Clin Pathol.

[23] Luo Y, Ren F, Liu Y, Shi Z, Tan Z, Xiong H (2015). Clinicopathological and prognostic significance of high Ki-67 labeling index in hepatocellular carcinoma patients: a meta-analysis. Int J Clin Exp Med.

[24] Orzalli MH, Kagan JC (2017). Apoptosis and necroptosis as host defense strategies to prevent viral infection. Trends Cell Biol.

[25] Davidson S, Crotta S, McCabe TM, Wack A (2014). Pathogenic potential of interferon alphabeta in acute influenza infection. Nat Commun.

[26] Dong LL, Liu L, Ma CH, Li JS, Du C, Xu S (2012). E-cadherin promotes proliferation of human ovarian cancer cells in vitro via activating MEK/ERK pathway. Acta Pharmacol Sin.

